# A series of workshops to present research findings and fill knowledge gap among adolescent girls and young women on sexual and reproductive health in Lebanon: An example of active knowledge dissemination

**DOI:** 10.7189/jogh.13.03044

**Published:** 2023-09-01

**Authors:** Rayan Korri, Olena Ivanova

**Affiliations:** 1 Division of Infectious Diseases and Tropical Medicine, Medical Centre of the University of Munich (LMU), Munich, Germany; 2 German Center for Infection Research (DZIF), Partner Site Munich, Munich, Germany

**Figure:**
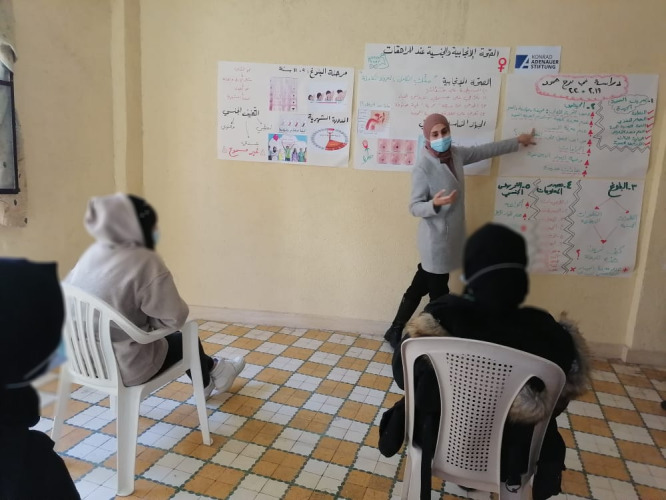
Photo: The doctoral researcher presenting the study findings of the research project “Sexual and Reproductive Health of Adolescent Refugee Girls and Young Women in Lebanon” to some of its participants, in one of the workshops designated for knowledge dissemination. The first author holds copyright of this photo.

Knowledge translation (KT) is a process that contains four steps: i) synthesis, ii) dissemination, iii) exchange, and iv) ethically sound application of knowledge [1]. It is an interplay between researchers as knowledge producers on the one hand and policymakers, practitioners, young people, or other stakeholders as knowledge users on the other hand [1]. Level of involvement, richness, and complicacy is based on the type of research, its results, and the unique necessities of every knowledge user group [2]. In case of health-related research, the aim of KT is to ameliorate individual and public health in addition to strengthen health systems and services [1]. Through dissemination, a fundamental step of KT, the researcher presents study results and recommendations to a specific audience using different means of communication [1-3]. That is not limited to publications in peer-reviewed journals and conference presentations, but also includes informative meetings with policymakers, practitioners, and study participants [1,4,5]. In case of low- and middle- income countries (LMICs), KT offers a mean through which inequalities in health issues might be minimised [6,7]. In order to effectively do that, KT approaches should be contextualised based on the particular cultural, social, financial, and political conditions of the setting where it is being performed [8,9]. The role of contextualised KT in decreasing health inequalities is even more significant when being carried out in the contexts of vulnerable groups such as asylum seekers, refugees, and migrants. In this article, we aim to highlight the importance of knowledge translation and dissemination with study participants on the one hand and share with the scientific community our experience and reflections when organising and delivering a series of interactive workshops for that purpose. Through the mentioned workshops, we presented the findings of our research project “Sexual and Reproductive Health of Adolescent Refugee Girls and Young Women in Lebanon” to its participants, who are Syrian adolescent refugee girls and young women residing in a Lebanese urban setting.

Knowledge dissemination depends on the characteristics of the actors involved in it, the study, and the selected approach of communication [10,11]. Different studies show that the means of knowledge dissemination vary in efficacy in terms of information delivery to users and their own comprehension of these information. A greater efficacy leads to a smoother and a more beneficial application of knowledge by users, irrespective of their types [12,13]. Passive dissemination methods, although being the main ones selected by researchers, show limited impact compared to active methods that include interaction and participatory methods. Accordingly, small group discussions and workshops are more productive in applying research-based guidance in comparison to publication of articles in scientific journals or distribution of informative texts [5,13,14]. It was found that only 60% of research institutions conduct activities on knowledge dissemination with a particular audience and just 50% of the same institutions dedicate sessions for discussions with them on research findings and significance [5].

## WHY IS IT IMPORTANT TO DISSEMINATE RESEARCH FINDINGS TO STUDY PARTICIPANTS?

It has been demonstrated that previous study participants as well as researchers consider knowledge dissemination as an essential and a beneficial step for both groups [15-18]. It is seen as a moral obligation of researchers which ensures that they do not primarily develop their profession and generate additional academic work through participants as cause of evidence, but also come back to the community to share relevant findings and thus add to the work done in the area of public health [15,16,18,19]. Moreover, dissemination enhances transparency and fosters a sense of involvement by study participants and their communities, which maintain their activity and commitment to the study in specific and research in general [16,20].

Participants believe that they own study findings, since they gave their consent for participation and offered the required data for that [18]. Through dissemination, participants find a recognition of their time, participation, and right to study results [15,16,18]. According to them, it should happen on participant and societal levels because of the possible positive impact of findings on personal and public health [18,21]. Presenting findings leads to a greater research quality considering that it builds a trustful relationship between researchers and participants on one hand and promotes future participation in further studies on another hand [18].

Since we believe that knowledge dissemination is a very important practice, we describe below an example on how it could be implemented. The outlined example shows how the knowledge dissemination of findings on a sensitive topic, such as sexual and reproductive health (SRH), can provide knowledge sharing and information gaps filling.

## KNOWLEDGE DISSEMINATION THROUGH A SERIES OF WORKSHOPS ON SEXUAL AND REPRODUCTIVE HEALTH IN BOURJ HAMMOUD, LEBANON

“Sexual and Reproductive Health of Adolescent Refugee Girls and Young Women in Lebanon” is the title of a doctoral research project that we conducted in Bourj Hammoud, an urban setting in Lebanon, between April 2018 and November 2021. The main goal of the project was to investigate and report the SRH status of Arab and Kurdish Syrian refugee adolescent girls and young women living in that setting. The exploratory study addressed that goal through two research approaches: i) a qualitative one, using focus group discussions, that examined the SRH perceptions and experiences of adolescent Syrian refugee girls aged 13 to 17 years, ii) a quantitative one, using a cross-sectional survey, that evaluated the SRH knowledge and access to services of young Syrian refugee women aged 18 to 30 years. We published our findings in peer-reviewed journals [22,23].

In January 2022, we organised and coordinated a series of eight workshops on various SRH topics in Bourj Hammoud. The workshops had two aims: i) to report back the study findings of the research project to the refugee adolescent girls and young women who participated in it; ii) to raise awareness among them on specific SRH issues based on the knowledge gap that they showed in the study. However, the workshops’ participants were not only limited to Syrian refugees but also included vulnerable Lebanese and other non-Lebanese girls and young women (individuals with lower socio-economic status) inhabiting Bourj Hammoud. A female Syrian gatekeeper living in Bourj Hammoud was responsible for recruiting the workshops’ participants. A total of 64 Lebanese, Arab Syrian, Arab Kurdish, Ethiopian, and Srilankese girls and young women participated in the workshops, which were held in Levantine Arabic.

The first round of four workshops was delivered to 32 married and unmarried young women from 18 to 35 years old, whereas the second round to 32 married and unmarried adolescent girls between 13 and 17 years old. All workshops took place in the gatekeeper’s apartment, where preventative measures for COVID-19 were followed. Childcare providers were present and stayed with the children of the participants in a separate room of the apartment in which they performed different educational activities. In order to ensure privacy, active participation, and group interaction, caregivers who accompanied the girls to the workshops were asked to either wait with the gatekeeper in a different room or to come back at the end of the workshop. The duration of every workshop ranged between 1.5 and 2 hours, depending on the engagement and questions of participants.

Each workshop started by presenting the key research findings of one study part, depending on the age group of the participants. Afterwards, an educator, who is also a medical doctor specialised in obstetrics and gynaecology with previous experience in conducting similar workshops, presented evidence-based information on five different themes. The doctoral researcher (Rayan Korri) and the educator decided on the themes based on the findings of the study and the shortage of SRH information identified through that. The themes discussed in the workshops for young women were: female reproductive system, menstrual cycle, sexual transmitted infections, methods of contraception, and pregnancy care; and for adolescent girls: definition of SRH, female reproductive system, puberty, menstruation, and sexual harassment. All participants had the chance to get involved in the discussions initiated by the educator in addition to asking questions. In the course of information presentation, there was short break to introduce and distribute sustainable menstrual products: reusable pads and menstrual cups. We presented their function, advantages and mode of application and gave practical tips on using them in an effective and hygienic way. Unmarried young women preferred to receive reusable pads instead of menstrual cups. Since the concept of menstrual cups was very new to all participants, we created a WhatsApp group with them in which educational videos on this reusable hygiene feminine product were shared in addition to a hotline provided by the supplier in Lebanon, through which women can ask their questions and share their concerns. The objective of this step was to support the adolescent girls and young women in fighting period poverty, an issue that is becoming crucial in Lebanon. In a country experiencing complex crises since 2019, the world’s worst economic crisis since the mid-19^th^ century being one of them, 78.43% of girls and women in Lebanon find difficulties in accessing menstrual hygiene products [24,25].

To see the positive impact and value of knowledge dissemination the participants were asked to answer a number of questions related to received information. For young women, we used five open-ended questions, which were answered in written form before and after every workshop. For adolescent girls, we asked questions to the entire group in an encouraging and interactive atmosphere to avoid the feeling to be graded or classified similar to schooling. Overall, we observed that the knowledge around different topics improved after the workshops. Doing this, we also got to know the false information that the participants still had after the workshops and the educator was able to correct them.

At the end of the workshops, many participants expressed their appreciation of having participated in the workshops, which they perceived as an opportunity of learning essential information. They also indicated their wish of having similar educative workshops more frequently. Different young women openly talked about their surprise and distress because of going through several reproductive experiences, while lacking fundamental SRH knowledge.

## REFLECTION ON THE EXPERIENCE OF ORGANISING AND DELIVERING SERIES OF KNOWLEDGE DISSEMINATION WORKSHOPS

After successfully publishing our second scientific paper from the same research project, it was very clear to us, as main researchers of the project, that active dissemination of findings to study participants is a practice that we would like to apply. The first reason for that was to fulfil our moral obligations towards the female Syrian community in Bourj Hammoud, while the second one was to reinforce transparency between the doctoral researcher on the one hand and the Syrian refugee adolescent girls and young women who participated in the study, their caregivers and social circles on the other hand. That was especially important for the doctoral researcher, since she was responsible of developing relationships with local actors and inhabitants, collecting data, and having daily direct contact with the Syrian refugee community during the fieldwork phase of the study.

The first step in our plan was to attract funding for the dissemination activities, which we successfully did from Konrad-Adenauer-Foundation Lebanon office. Although our dissemination plan did not require a large budget, receiving financial support was very important in order to cover the different expenses of the eight workshops. As second step, we contacted one of the female Syrian gatekeepers who were involved in our research project. She recruited again the study participants, explained to them the goal of the workshops, and offered her apartment as a safe and private space of knowledge exchange on SRH issues. We believe that knowledge dissemination on a sensitive topic such as SRH and to a vulnerable target group such as refugee adolescent girls and young women might be very challenging and with limited effectiveness if not involving a gatekeeper from the same community. It was key to receive again trust on the level of the Syrian refugee female community in Bourj Hammoud, especially because of time gap between exiting the field after finalising data collection (March 2020) and re-entering it for knowledge dissemination (January 2022).

Finally, we would like to reflect on some practical points that were taken into consideration when preparing for the dissemination workshops and presenting the research findings to the study participants. It was crucial to be constantly aware of our audience´s particularity and the approach through which we want to deliver the information to that audience. In our workshops, the participants did not have a scientific or an academic background, in contrast to what we are familiar with in terms of communication and exchange, but a conflict-affected group experiencing an enhanced vulnerability. In order to explain the goal of the workshop and the findings of our research project to them in a simplified and understandable way, no specialised terminologies were used but easy Levantine Arabic ones that the adolescent girls and young women themselves adopt when exchanging SRH information and experiences in their own communities. Furthermore, we summarised and rephrased our research findings to be presented in a comprehensive way without focusing on statistical or thematic analysis. The doctoral researcher reported the various findings while highlighting the ones on SRH knowledge and sources of information and keeping a certain sequence of ideas and examples, which allowed a smooth flow of information. This flow was further endured and supported by the educator, when raising awareness on specific SRH topics.

## CONCLUSIONS

Knowledge dissemination has different advantages such as improving the quality and transparency of a study as well as the engagement of participants and their social circles in research. It is considered a significant practice by participants and their communities, since it benefits individual and public health. We highly recommend interactive workshops with study’s participants, similar to the ones that we have organised and coordinated for our study on the “Sexual and Reproductive Health of Adolescent Refugee Girls and Young Women in Lebanon”, as a method for active and effective knowledge dissemination.
